# Facile Preparation of Cellulose Bioplastic from *Cladophora* sp. Algae via Hydrogel Method

**DOI:** 10.3390/polym14214699

**Published:** 2022-11-03

**Authors:** Steven Steven, Anna Niska Fauza, Yati Mardiyati, Sigit Puji Santosa, Silvia Mar’atus Shoimah

**Affiliations:** 1 Materials Science and Engineering Research Group, Faculty of Mechanical and Aerospace Engineering, Institut Teknologi Bandung, Jl. Ganesha 10, Bandung 40132, Indonesia; 2 Lightweight Structure Research Group, Faculty of Mechanical and Aerospace Engineering, Institut Teknologi Bandung, Jl. Ganesha 10, Bandung 40132, Indonesia

**Keywords:** cellulose, bioplastic, biodegradable material, *Cladophora* sp. algae, hydrogel method, facile process

## Abstract

Bioplastic has been widely studied in the past decades as a replacement for non-biodegradable and non-environmentally friendly plastic. One of the promising materials to produce bioplastic is cellulose. However, it is rarely used as the main component for bioplastic production. This study reports a facile process to prepare bioplastic using the pure cellulose content of *Cladophora* sp. algae via the hydrogel method. The effect of epichlorohydrin (ECH) concentrations as the cross-linking agent was investigated toward the biodegradability, thermal, and mechanical properties of the cellulose bioplastic obtained. The results showed that ECH concentrations affected the properties of the cellulose bioplastic produced due to the number of cross-links formed during the process. The cellulose bioplastic possessed relatively high thermal and mechanical properties. The cellulose bioplastic performed excellent biodegradability, as it was degraded by more than 40% within five days. Thus, the cellulose of *Cladophora* sp. algae has the potential to be developed as the main component for bioplastic application.

## 1. Introduction

Plastic is a rapidly growing material in the industry; it has become an inseparable product and improves the standard of living of human beings. Unfortunately, most plastics are disposable products that end up in the landfill, soil, and marine environment [1]. Recently, bioplastics have been developed as an eco-friendly plastic material with excellent properties, such as being biodegradable, non-toxic, and having a lower carbon footprint than conventional plastics. Bioplastics can be made from renewable resources or organic waste [2,3]. Among the renewable resources, cellulose is one of the promising materials to be investigated for use as bioplastics, owing to its abundance and excellent properties. Cellulose is one of the natural polymers extracted from various plants [4]. It consists of β(1–4)-linked d-anhydroglucopyranose units, forming a long linear chain packed densely due to the formation of intra- and intermolecular hydrogen bonds, which generates outstanding mechanical properties [5,6]. In addition, the structure of cellulose has the ability to be modified for advanced applications [7]. 

The preparation of cellulose bioplastics has been extensively developed in recent years. The cellulose was obtained from various resources such as oregano waste [8], cotton and sugarcane bagasse [9], rice straw [10], and many others. However, cellulose was merely used as filler or modified as a cellulose derivative in the preparation of bioplastics. The most commonly used cellulose derivative is cellulose acetate, which has excellent mechanical and thermal properties. However, it requires a complicated and complex process [8,11]. As a filler, cellulose has been added to various matrices such as amylose [12], carrageenan [13], and starch [14]. The results showed that cellulose improved the properties of bioplastics. Nevertheless, the utilization of cellulose as the main component of bioplastics has not been widely studied. 

One of the alternative methods to produce bioplastics with cellulose as the main components without any modification process is the hydrogel method. Recently, the preparation of cellulose hydrogel has been broadly studied and reported. One of the studies on cellulose hydrogel was by Navarra et al., who investigated the fabrication of cellulose hydrogel and ECH for energy storage. The result showed that hydrogel had good absorption characteristics on the electrolyte solution [15]. Another study by Cui et al. reported the investigation of cellulose hydrogel for a non-toxic and flexible electronic application. The study revealed a decrease in the mechanical properties of hydrogel along with an increase in the cross-linking agent (ECH). Nevertheless, the hydrogel was able to be fully degraded in 28 days [16]. A previous study by Chang et al. also observed the preparation of cellulose hydrogel with two different post-treatments, i.e., the heating and freezing method. The result indicated that the obtained hydrogel through heating post-treatment exhibited better transparency than the freezing method [17]. Alam et al. also successfully prepared cellulose hydrogel-ECH as superabsorbent polymers (SAP) in their study [18]. However, apart from these prior studies, reports on the preparation of cellulose bioplastic through the hydrogel method are still extremely limited. On the other hand, Sun et al. successfully prepared bioplastic from κ-carrageenan through the hydrogel method and processed it into a transparent film for packaging application [19]. Therefore, the previous study encouraged this research on using cellulose as the main component via the hydrogel method for bioplastic application.

Cellulose exhibits certain properties and characteristics depending on its resources [20]. *Cladophora* sp. algae is among the potential resources of cellulose with unique properties that have gained numerous interest. This species of green filamentous algae grows uncontrollably in both brine and freshwater, polluting the surrounding environment, as it rapidly forms dense mats on the water surface. This can harm the marine biota, since it blocks the sunlight and reduces the oxygen supply for the living organisms in the water. Therefore, the utilization of *Cladophora* sp. algae cellulose is expected to overcome this problem. The cellulose content in Cladophora sp. algae is around 20–30%. The cellulose has a high degree of crystallinity, which is almost 95% [21,22]. *Cladophora* sp. algae cellulose has a web-like structure where the fibrils exhibit a highly entangled network. This structure generates cellulose with high mechanical properties [23]. Hence, the cellulose extracted from *Cladophora* sp. algae has the potential to produce bioplastics with excellent characteristics compared to other cellulose resources. 

In this study, the cellulose hydrogel from *Cladophora* sp. algae was prepared for bioplastics preparation. Cellulose hydrogel is commonly prepared in two steps, i.e., dissolution and cross-linking process. Firstly, cellulose is dissolved using a certain solvent. Alkali/urea is the most widely used solvent for hydrogel due to its rapid dissolution, simplicity, eco-friendliness, low toxicity, and low cost [24]. A stable-structured cellulose hydrogel can be obtained through a chemical cross-linking reaction. Epichlorohydrin (ECH) is largely chosen as the cross-linking agent for this process. The cross-link is controlled by the etherification reactions between the hydroxyl groups of cellulose–ECH and the entanglement of cellulose fibrils under alkaline conditions [25]. Thereby, *Cladophora* sp. algae cellulose was dissolved in NaOH/urea/water solution, while ECH was used as the cross-linking agent to form the cellulose hydrogel. The effects of ECH concentrations on the characteristics of the obtained bioplastic, such as its mechanical, thermal, and biodegradable properties, were carefully studied. This research focused on utilizing *Cladophora* sp. algae pollution (natural waste) as a cellulose resource and the preparation of cellulose bioplastics with the hydrogel method as a facile preparation process.

## 2. Materials and Methods

### 2.1. Materials

*Cladophora* sp. algae used in this study were acquired from Krakal Beach, Yogyakarta, Indonesia. Sulfuric acid (H_2_SO_4_) 95–97% was supplied by Smart-Lab Indonesia (Tangerang, Indonesia). Epichlorohydrin (ECH) was purchased from Merck (Darmstadt, Germany). Sodium hydroxide (NaOH) and hydrogen peroxide (H_2_O_2_) were obtained from Bratachem Inc. (Bandung, Indonesia). Urea was purchased from Central Kimia Bandung (Bandung, Indonesia).

### 2.2. Preparation of Cellulose Bioplastic

#### 2.2.1. Cellulose Extraction

The isolation of *Cladophora* sp. cellulose was carried out by conducting alkalization, acid hydrolysis, and bleach treatment. *Cladophora* sp. algae were dried and then cleaned with washing detergent and tap water to remove all dirt. Then, they were left at room temperature to dry. Subsequently, the algae were alkalized with 17.5 wt% NaOH in a reflux condenser system (Pyrex, Glendale, AZ, USA) at 100 °C for 3 h. The obtained residue was washed with tap water to reach its neutral pH and dried at room temperature. Afterward, the alkalized *Cladophora* sp. algae were treated in acid of 1 M H_2_SO_4_ in the reflux condenser system at 100 °C for 3 h. Then, the residue was washed with water until its pH was neutral and then dried at room temperature. Furthermore, the acid-treated *Cladophora* sp. algae were bleached in the reflux condenser system with 5 wt% H_2_O_2_ at 100 °C for another 3 h treatment. The final residue was then washed and dried at room temperature. The obtained cellulose of *Cladophora* sp. algae was in a form of solid paper.

#### 2.2.2. Cellulose Dissolution

An amount of 1 wt% of *Cladophora* sp. cellulose was cut into the size of 1 × 1 mm^2^ and then dissolved in NaOH/Urea/water solvent solution with the concentration of 7 wt% of NaOH/12 wt% of urea pre-cooled in a freezer for 24 h. The mixture of cellulose and solvent solution was stirred in a magnetic stirrer (Pyrex, Glendale, AZ, USA) for 5 h, with the temperature being kept by an ice-bath cooling system. After being stored in a freezer overnight, the solution was stirred for another 5 h in the ice-bath cooling system so that the cellulose was well-dissolved. The final solution was stored in a freezer for further processing.

#### 2.2.3. Cellulose Bioplastic 

In this study, the cellulose bioplastics were prepared by applying the hydrogel method. For this purpose, ECH in different concentrations (10; 15; 20; 25 wt%) were added into the cellulose solutions as prepared in Section 2.2.2. Each of them was then stirred in a magnetic stirrer for 90 min at room temperature. Afterward, the mixture was molded in a plastic container and kept overnight at room temperature until the hydrogel was formed. The attained hydrogel was immersed in water to remove excess ECH, NaOH, and urea. The water for soaking the hydrogel was changed three times a day until a neutral pH was reached. Furthermore, the hydrogel was dried at room temperature to obtain the cellulose bioplastics. The resulting cellulose bioplastic samples were labeled according to the increment of ECH concentrations as follows: C-ECH10, C-ECH15, C-ECH20, and C-ECH25.

### 2.3. Characterizations

#### 2.3.1. Fourier Transform Infrared Spectroscopy

The functional groups of the cellulose bioplastics were identified and characterized by using Fourier Transform Infrared Spectrophotometry (FTIR Prestige 21, Shimadzu Corporation, Kyoto, Japan). The samples and KBr were pressed with a pressure of 10 tons to form pellets. The samples were placed in a sample holder and scanned in the range of 4000–600 cm^−1^.

#### 2.3.2. Thermogravimetric Analysis

The thermal stability of the cellulose bioplastics was examined using thermogravimetric analysis (TG/DTA Hitachi STA7300, Hitachi High-Tech Corporation, Tokyo, Japan). For this analysis, the samples were cut into the size of 2 × 2 cm^2^ and placed on an aluminum pan. The measurement was performed in a nitrogen atmosphere with a heating rate of 10 °C min^−1^ (30–600 °C).

#### 2.3.3. Tensile Test

The tensile properties of the cellulose bioplastics were determined by utilizing Tensilon RTF-1310 (A&D Company, Tokyo, Japan), according to the ASTM D882 standard. The samples were prepared in a size of 6.25 × 1.25 cm^2^ and the test was conducted with a speed test of 6.25 mm.min^−1^.

#### 2.3.4. Water Contact Angle Measurement

The wetting characteristic of cellulose bioplastics was determined by measuring the water contact angle on the flat surface of cellulose bioplastics. The measurement was conducted using a digital microscope (Chongqing Dontop Optics Co., Ltd., Chongqing, China). An amount of 10 µL of water was dropped onto the surface of the samples at room temperature. The image was captured and then the water contact angle was measured using ImageJ software (ImageJ 1.52a, Wayne Rasband, National Institute of Health, Bethesda, MD, USA). 

#### 2.3.5. Solubility in Water Test

The solubility in water of the cellulose bioplastics was analyzed by immersing the sample in the water. The samples prepared in 1.5 × 1.5 cm^2^ were dried in an oven at 60 °C for 24 h. Firstly, the samples were weighed for the initial weight (W_1_) using digital balance, placed in a beaker glass filled with 50 mL of DI water, and then left at room temperature for 24 h. The samples were subsequently dried in an oven (IKA, Petaling Jaya, Malaysia) at 60 °C for another 24 h. The final samples were weighed for their final weight (W_2_) using a digital balance. The percentage of solubility in water was calculated using Equation (1):

(1) $$Solubility\;in\;water\;\left(\%\right)=\frac{\left(W_{1}-W_{2}\right)}{W_{1}}\times 100$$

#### 2.3.6. Biodegradation Test

A biodegradation test of the cellulose bioplastics was conducted by applying the soil burial method according to the previous study [26]. The samples were prepared in 1.5 × 1.5 cm^2^ and W_1_ of each sample was weighed using a digital balance. To carry out the test, the samples were buried in garden soil which was placed in Styrofoam cups. Afterward, the samples were kept in the cups for five days with controlled moisture of the soil at room temperature. Finally, the samples were removed from the soil, washed with water, and dried in an oven at 60 °C for 24 h. The W_2_ was weighed using a digital balance. The biodegradation percentage was calculated using Equation (2):

(2) $$Biodegradation\;\left(\%\right)=\frac{\left(W_{1}-W_{2}\right)}{W_{1}}\times 100$$

## 3. Results

### 3.1. The Preparation of Cellulose Bioplastic

**Figure 1:**
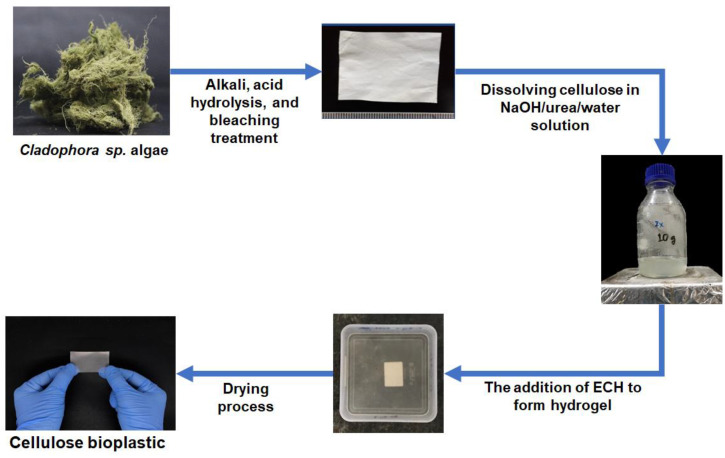
The scheme of the cellulose bioplastics preparation from *Cladophora* sp. algae.

The cellulose was extracted from *Cladophora* sp. algae with a yield of ±28% and a purity of ±97%. Then, the extracted cellulose was utilized for cellulose bioplastic material. The cellulose bioplastics were successfully prepared from *Cladophora* sp. algae via the application of the hydrogel method, as shown in Figure 1.

**Figure 2:**
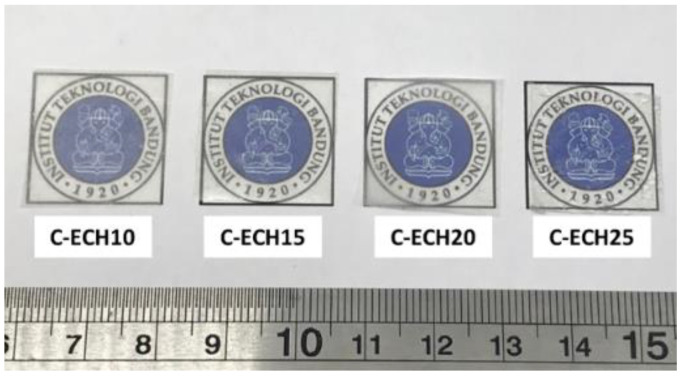
Cellulose bioplastic samples at various ECH concentrations.

In this study, *Cladophora* sp. algae cellulose was dissolved in NaOH/urea/water solution. ECH at various concentrations was used as the cross-linking agent to form the hydrogel. During the preparation of the cellulose hydrogel, the mixture of cellulose solution and ECH exhibited an exothermic reaction due to the heat produced during the gelation process. Cellulose hydrogel was formed overnight after some rest time through the chemical cross-linking between the hydroxyl groups of cellulose and ECH. According to Ciolacu et al., the chain entanglements of cellulose were also formed during this process [25]. In order to remove the chemical residue, the cellulose hydrogel was immersed in water until its neutral pH was reached. Finally, cellulose bioplastics were achieved after the process of drying the cellulose hydrogels. The obtained cellulose bioplastic samples at various ECH concentrations are presented in Figure 2. The cellulose bioplastic samples were placed with a background of Institut Teknologi Bandung’s logo. According to the visual inspection, the optical clarity of cellulose bioplastics increased with the increase in ECH concentrations. The C-ECH10 sample exhibited the highest opacity compared to the other bioplastic samples. The opacity of cellulose bioplastic is affected by the cellulose concentration in the bioplastic, which is related to the tendency of cellulose molecules to form aggregates. The aggregates were caused by the hindrance of intermolecular bonding within the cellulose molecules. Furthermore, the previous study also showed similar behavior to the present study [13]. 

The data of the sample with 5% ECH addition could not be present in this work due to its inability to form a hydrogel during the cellulose bioplastic preparation. Therefore, only the samples with 10–25% ECH addition are presented in this report. 

### 3.2. Chemical Structure of Cellulose Bioplastic

**Figure 3:**
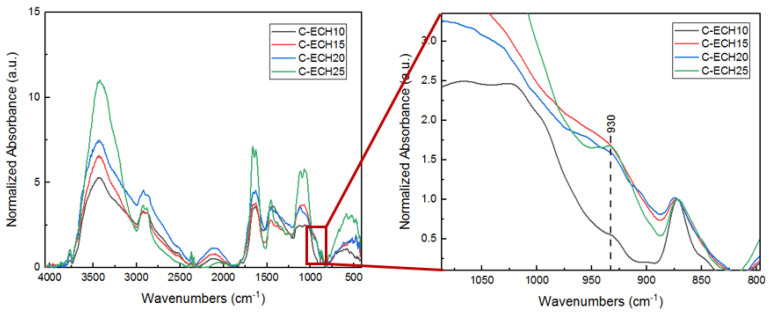
FTIR spectra of cellulose bioplastics at various ECH concentrations.

The FTIR spectra of cellulose bioplastics at various concentrations can be seen in Figure 3.

The FTIR spectra of the cellulose bioplastics were normalized at 872 cm^−1^, which indicated the characteristic of β-d-glycosidic linkage. The cross-linking between cellulose and ECH was confirmed by the formation of ether bonds (C–O–C) at 1067 cm^−1^. The FTIR spectra of cellulose bioplastics at various ECH concentrations showed that the formation of C–O–C (ether bonds) increased along with the increase in ECH concentrations. It was confirmed through an increase in the peak ratio of ether bonds at 1067 cm^−1^ to β-d-glycosidic linkage at 872 cm^−1^. Moreover, the peaks at 1116 cm^−1^ and 3420 cm^−1^ also increased with the increase in ECH concentrations, which were assigned to C–O–C and O–H vibrations, respectively. The hydroxyl groups increased in number due to the greater amount of ether bonds formed that had prevented the cellulose chains entanglement, which caused more hydroxyl groups to be free in vibration mode. The result was also confirmed in a previous study by Cui et al. and Zhao et al., which reported that C–O–C vibrations were assigned at 1161 cm^−1^, C–O stretching at 1022 cm^−1^ and 1030 cm^−1^, and C–O–C in β-d-glycosidic linkage at 896 cm^−1^ [16,27]. Furthermore, cellulose bioplastic with 25 wt% of ECH concentration showed a slightly different peak at 930 cm^−1^, which was attributed to the C–C symmetric vibrations of oxirane in the ECH structure [28]. This is possibly an indication of unreacted ECH in the cellulose bioplastics.

### 3.3. Thermal Stability

**Figure 4:**
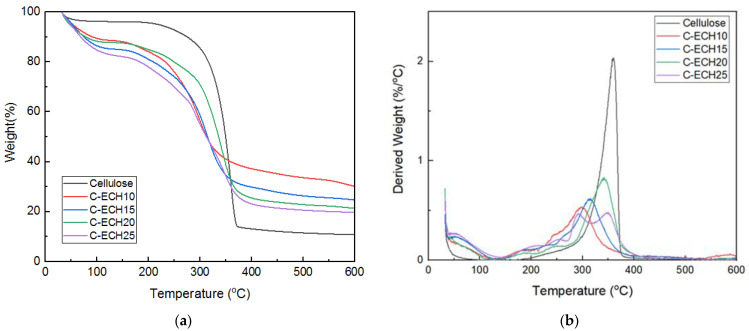
(**a**) TG and (**b**) DTG analysis of the cellulose and cellulose bioplastic samples at various ECH concentrations.

The thermal stability of the cellulose and cellulose bioplastics was analyzed using thermogravimetric analysis (See Figure 4). The results showed that the cellulose bioplastics had a relatively good thermal stability at high temperatures. The cellulose bioplastics at various ECH concentrations had a similar degradation behavior with several stages of weight loss. The cellulose bioplastic experienced a weight loss of ±20% in the range of 80–230 °C. This is possibly due to the evaporation of moisture content and the remaining unreacted ECH. Moreover, the ECH compound is slightly soluble in water, which evaporates simultaneously with the moisture content [24]. Then, significant weight loss occurred in the range of 230–370 °C. The cellulose bioplastics exhibited a lower degradation temperature than pure cellulose due to the amorphous structure that formed. In the range of 370–600 °C, cellulose material decomposed to form a polycyclic aromatic ring compound, which is indicated as char. The char is composed of carbon content which remains as a solid by-product after thermal decomposition [29,30]. In this case, char production decreased with the increase in ECH concentrations. The cellulose bioplastic with high ECH concentration resulted in a high degree of cross-linking bonds. This presumably affected the atomic ratios of oxygen/carbon within the chemical compound of bioplastic. Therefore, it resulted in a low yield of char. In addition, the amount of water content and unreacted ECH increased with the increase in ECH concentrations. It resulted in a decrease in cellulose content and decreased char formation compared to the other samples.

### 3.4. Mechanical Properties

**Figure 5:**
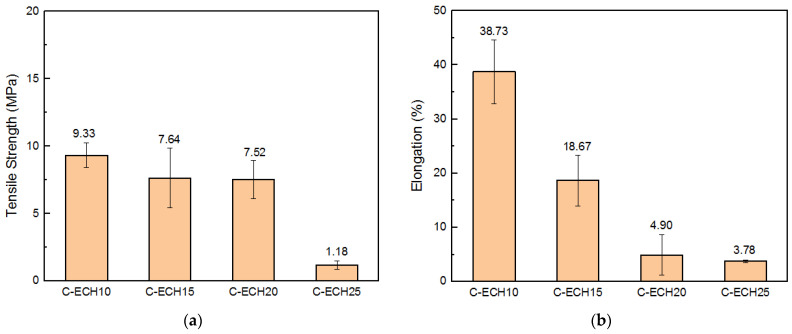
(**a**) Tensile strength and (**b**) elongation of the cellulose bioplastic samples at various ECH concentrations.

The tensile properties of the cellulose bioplastics at various concentrations of ECH are presented in Figure 5. The cellulose bioplastics did not show significant differences in tensile strength with the increase in ECH concentrations. The highest tensile strength obtained was 9.33 MPa. The result indicated that the tensile strength is comparable to other bioplastic types, such as cellulose acetate, cellulose/carrageenan, and chitosan [11,13,31]. However, the cellulose bioplastic with 25% ECH concentration declined rapidly to 1.18 MPa. This possibly occurred due to the unreacted ECH in the cellulose bioplastics, which was confirmed by the exhibited peak in the FTIR spectrum at 930 cm^−1^, as shown in Figure 3.

The elongation of cellulose bioplastics decreased as the ECH concentrations increased. The highest percentage of elongation was 38.73%, with the addition of 10 wt% of ECH concentration to the cellulose molecules. The increase in ECH concentration resulted in a higher cross-link formation within the cellulose molecules. Consequently, the rearrangement of cellulose chains was obstructed, and a greater number of amorphous regions were formed. The amorphous region is responsible for the elongation of cellulose bioplastics. However, the chain motion in the amorphous regions was restricted at a higher degree of cross-links within the cellulose molecules. Hence, the elongation of cellulose bioplastic decreased. In addition, the increase in ECH concentrations also increased the water uptake of the cellulose molecules. It resulted in the formation of a great number of micropores. The micropores caused stress concentration during the tensile test and reduced the elongation of the cellulose bioplastic.

**Figure 6:**
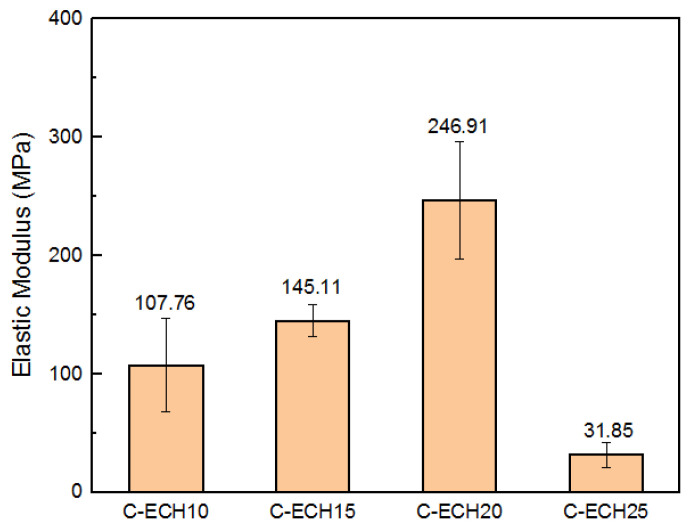
Elastic modulus of the cellulose bioplastic samples at various ECH concentrations.

The elastic modulus of the cellulose bioplastics is shown in Figure 6, where it is seen to experience an increase along with the addition of ECH concentration from 10 to 20 wt%. The present study showed a higher elastic modulus compared to the cellulose acetate bioplastic from the previous study, which was 246.91 MPa and 153 MPa, respectively [11]. However, the elastic modulus significantly decreased in the sample with 25 wt% of ECH concentration. The bioplastic sample with 25% ECH concentrations experienced the least elastic modulus compared to the other samples. This is presumably due to the excess formation of micropores and defects sites [32], which resulted in stress concentrations during the tensile test. Therefore, it decreased its elastic modulus.

### 3.5. Solubility in Water and Wetting Characteristic of Cellulose Bioplastic

**Figure 7:**
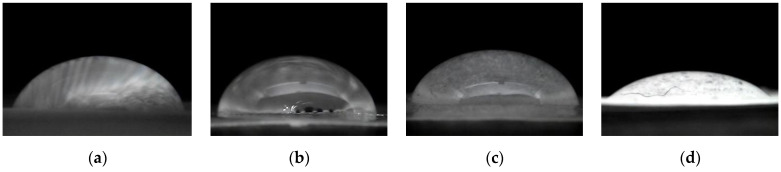
Water contact angle of (**a**) C-ECH10, (**b**) C-ECH15, (**c**) C-ECH20, (**d**) C-ECH25 samples.

The wetting characteristic of the cellulose bioplastics was determined by applying the water contact angle method, as seen in Figure 7.

Cellulose bioplastic samples with 10, 15, and 20 wt% of ECH concentrations exhibited a similar contact angle to one another, while the cellulose bioplastic sample with 25 wt% of ECH concentration experienced a significant decrease by 50%. The water contact angle value of each sample is shown in Table 1. A greater amount of ECH concentration added to the cellulose bioplastic increased the free volume between the cellulose chains. The increment of the formed cross-links hindered the formation of crystalline regions of the cellulose chains. Therefore, more hydroxyl groups were exposed to interact with water and lower the contact angle value.

**Figure 8:**
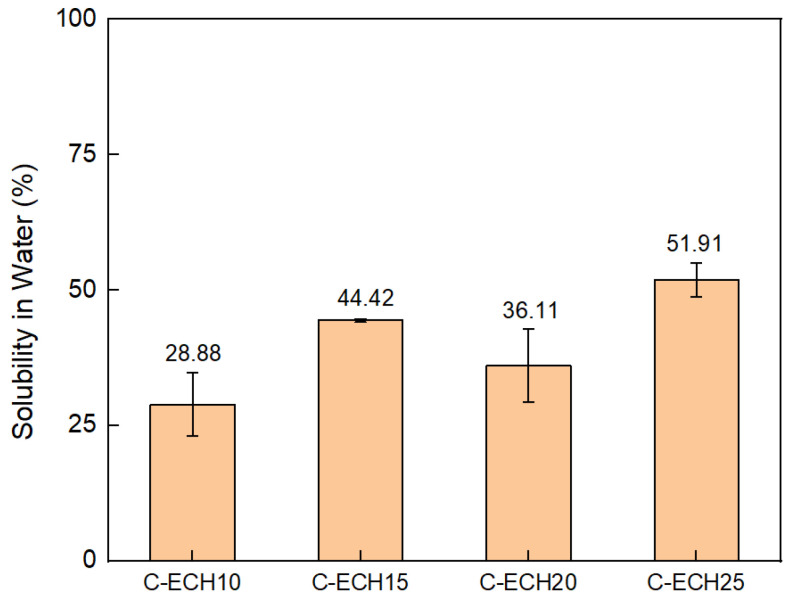
Percentage of the solubility in water of the cellulose bioplastic samples at various ECH concentrations.

The percentage of water solubility of the cellulose bioplastics is presented in Figure 8. As the concentration of ECH increased, the cellulose bioplastic became more soluble in water. This was due to the lower crystallinity of cellulose–ECH structure generated at the sample with higher ECH concentrations. For the sample with a lower concentration of ECH, the number of hydrogen bonds between the cellulose chains was greater. This prevented the solubility of the cellulose bioplastic in water. In addition, the excess formation of cross-linking bonds at high ECH concentration increased the free volume within the cellulose molecules. Therefore, it also increased the water uptake of cellulose bioplastic. The result is also shown in the previous study [32].

### 3.6. Biodegradability

**Figure 9:**
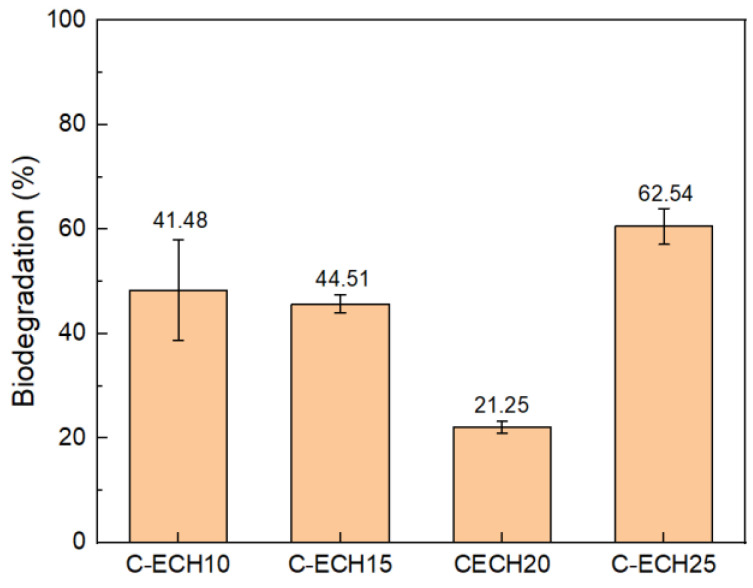
Percentage of biodegradation of the cellulose bioplastic samples at various ECH concentrations.

The biodegradation of the cellulose bioplastics was determined by conducting the soil burial method. The results of this process can be seen in Figure 9. The cellulose bioplastic samples relatively showed an excellent biodegradation. The cellulose bioplastics degraded by ±40% of weight loss in 5 days. The result obtained showed a higher percentage of biodegradation compared to cellulose/naringin bioplastic, where the weight loss occurred after 30 days of exposure in a seawater environment [33]. With the increase in ECH concentrations, the percentage of biodegradation decreased. This is possibly due to the formation of a high degree of cross-linking bonds, which prevents a rapid biodegradation mechanism. However, the biodegradation of cellulose bioplastic with a 25% ECH concentration increased. It was presumed by the excess formation of free volume or micropores between cellulose molecules [32]. Thus, the micropores increased the access sites for water and microorganisms, which led to a higher percentage of biodegradation.

According to the results above, the addition of ECH concentrations affected the formation of cross-link bonds in the cellulose molecules. The formation of cross-linking bonds by ECH was confirmed through FTIR spectra. In addition, the degree of cross-link affected the properties of the cellulose bioplastics, such as biodegradability, mechanical properties, optical clarity, water contact angle, and water solubility. The cellulose bioplastic possessed good thermal stability and mechanical properties, which were comparable to conventional plastics. Amongst all the ECH concentrations, the optimum cellulose bioplastic obtained was the 10% ECH concentration compared to the other samples.

## 4. Conclusions

In this research, we successfully prepared bioplastic from Cladophora sp. algae cellulose via the hydrogel method, which showed a facile preparation process. According to the result obtained, the optimum ECH concentration used for cellulose bioplastic preparation was 10%, as it exhibited good biodegradability by more than 0% within five days and the least water solubility. Moreover, the mechanical properties of the cellulose bioplastic were comparable with the commodity plastics used, with a tensile strength of 9.33 MPa, elongation of 38.73%, and elastic modulus of 107.76 MPa.

The beneficial impact of this study includes: (1) The cellulose was used as the main component for bioplastic production, while the other studies mostly used modified cellulose, or it was used as a filler prior to bioplastic production. (2) The cellulose resource was obtained from biomass waste, which is harmful to its environment. Therefore, it helps the remediation of the current issue. (3) Cellulose was shown to be a promising material as the main component for bioplastic production with good performances for further studies.

## Figures and Tables

**Table 1:** Water contact angle of cellulose bioplastic.

Sample	Contact Angle (◦)
C-ECH10	71.31
C-ECH10	72.54
C-ECH20	72.92
C-ECH25	36.27

## Data Availability

Not applicable.
